# A case of brucellosis concomitant with HIV infection in China

**DOI:** 10.1186/s40249-020-0624-7

**Published:** 2020-01-16

**Authors:** Shuai-Bing Dong, Li-Ping Wang, Chao-Xue Wu, Fan Li, Yong Yue, Dong-Ri Piao, Hong-Yan Zhao, Hai Jiang

**Affiliations:** 1 Division of Infectious Disease, Key Laboratory of Surveillance and Early Warning on Infectious Disease, Chinese Center for Disease Control and Prevention, Beijing, China; 2 Sichuan Center for Disease Control and Prevention, Chongqing, China; 3 Chengdu Center for Disease Control and Prevention, Chengdu, China; 4 State Key Laboratory for Infectious Disease Prevention and Control, Collaborative Innovation Center for Diagnosis and Treatment of Infectious Diseases, Chinese Center for Disease Control and Prevention, National Institute for Communicable Disease Control and Prevention, Beijing, China

**Keywords:** Brucellosis, HIV, Pyrexia of unknown origin, Joint examination

## Abstract

**Background:**

Human brucellosis is a neglected public health issue in China and reports of HIV-infected individuals complicated with brucellosis are rare. This report describes the case of an HIV-infected patient complicated with brucellosis. We want to raise awareness of clinical diagnosis of brucellosis among clinicians. Furthermore, we should be more concerned about cases with pyrexia of unknown origin, especially in non-epidemic areas of brucellosis in China.

**Case presentation:**

We encountered the case of a 31-year-old HIV-infected male with a CD4+ T lymphocyte count of approximately 300. On May 1, 2019, the patient had onset of non-specific caustic irregular fever with body temperature reaching 41.0 °C. He was admitted to two medical institutions in Yunnan with pyrexia of unknown origin. Finally, on day 7 of hospitalization in the Public Health Clinical Medical Center in Chengdu City, he was diagnosed as having brucellosis infection based on blood culture results.

**Conclusions:**

This is the first reported case of brucellosis concomitant with HIV infection in China. Laboratories in infectious disease hospitals and category A level III hospitals in the southern provinces of China should be equipped with reagents for clinical diagnosis of brucellosis and to strengthen the awareness of brucellosis diagnosis in China. Secondly, in provinces with a high incidence of AIDS and brucellosis such as Xinjiang and Henan, it is recommended to implement a joint examination strategy to ensure the early detection, diagnosis, and treatment of this infection.

## Background

Brucellosis is a zoonotic bacterial infection caused by *Brucella* spp., and can be transmitted to humans from cattle, sheep, goats, and pigs via direct contact with the infected animals or through consumption of unpasteurized animal products [1, 2]. Human brucellosis can range from asymptomatic infections to severe symptoms with fever, fatigue, loss of appetite, and joint muscle and back pain [3]. The presence of clinical symptoms and epidemiologic risk factors for *Brucella* infection with a titer of 1:100 or higher as measured by the serum agglutination test (SAT) is considered a confirmed case of brucellosis [4]. Persons with laboratory-confirmed brucellosis are treated with rifampin and doxycycline for 6 weeks. Delayed diagnosis, inappropriate treatment, or failed treatment adherence can lead to chronic infections. Chronic infections involve complex treatment and can last for several years [5]. AIDS is an immune deficiency syndrome caused by the human immunodeficiency virus (HIV). As the immune systems of HIV-positive individuals are damaged, some researches have reported that they are more likely to be infected with brucellosis [6–8]. However, many studies in China have reported that in cases of HIV infection complicated with tuberculosis, but cases of HIV infection complicated with brucellosis have been rarely reported. Therefore, here we reported the case of an HIV-positive individual with brucellosis, and aimed to improve the brucellosis diagnosis awareness among clinicians, who should be particularly alert when encountering cases of unexplained fever, especially when these cases originate from non-epidemic areas of brucellosis in China.

## Case presentation

We presented the case of a 31-year-old male who had acquired HIV infection through unprotected sex with another man in 2016. He had a CD4+ T lymphocyte count of approximately 300, and had been prescribed antiretroviral therapy (ART) from 2016. On May 1, 2019, he had non-obvious caustic irregular fever and occasional chills, with his body temperature reaching 41.0 °C. The patient then went to a privately operated clinic for 2 days and received unknown medical infusion in Kaiyuan City, Yunnan Province, China. There was no improvement in his condition after 2 days, on May 3, 2019, and he was consequently referred to Kaiyuan People’s Hospital, where a routine blood examination was conducted. The laboratory results showed low leukopenia, low thrombocytopenia, and low hypokalemia. The patient was prescribed cephalosporin, hypothermia, and treatment to replenish fluids for 3 days, but the fever persisted.

On May 7, 2019, the patient returned to Chengdu from a business trip in Yunnan. He was hospitalized in Chengdu Center for Disease Control and Prevention on the same day. He received ART and biapenem anti-infective treatment for fever, chills, and bloodstream infection. The next day, he underwent a color Doppler ultrasound examination and the results of the scan revealed enlarged spleen, enlarged bilateral cervical lymph nodes, and enlarged bilateral inguinal lymph nodes. The routine blood examination revealed that he had neutropenia (Table 1). The patient continued to receive ART and biapenem anti-infective treatment during this period.

**Table 1:** Summary of the clinical biochemical testing

Sample type	Clinical indicators	Laboratory data	Unit	Reference range
Whole blood	Neutrophil count	1.99^a^	10^9^/L	2.00–7.00
	Neutrophil rate	38.2^a^	%	50.0–70.0
	Lymphocyte count	2.73	10^9^/L	0.80–4.00
	Lymphocyte rate	52.2^b^	%	20.0–40.0
	White blood cell count	5.22	10^9^/L	3.50–9.50
	Monocyte count	0.41	10^9^/L	0.12–1.2
	Monocyte rate	7.9	%	3.0–12.0
	ESR blood sedimentation	39^b^	mm/h	0–21
	CD3+ count	2648^b^	cells/μl	770–2041
	CD3 + CD4+ count	467	cells/μl	414–1123
	CD3 + CD8+ count	1523^b^	cells/μl	238–874
	CD3+ percentage	88.98^b^	%	6–82
	CD3 + CD4+ percentage	15.69^a^	%	40–58
	CD3 + CD8+ percentage	51.17^b^	%	15–32
	Lymphocyte count	2976	cells/μl	–
	Lymphocyte percentage	40.65	%	–
	CD4/CD8 ratio	0.31	–	–
Serum	Alanine aminotransferase	131^b^	U/L	0–37
	Aspartic aminotransferase	109^b^	U/L	0–37
	Transglutaminase	86^b^	U/L	0–50
	Hypersensitive C-reactive protein	82.9^b^	mg/L	0–5.0

^a^Laboratory data are below the reference range. ^b^Laboratory data are above the reference range - no data

On May 11, clinical laboratory reports confirmed the presence of gram-negative bacilli in blood culture, which was considered to be co-infected with severe immunodeficiency infection. The smear tested positive (+++) for acid-fast bacilli. This was initially suspected as tuberculosis infection, but the tuberculosis infection was ruled out by the X-ray check. Two days later, blood culture confirmed Malta *Brucella*. An epidemiological investigation was conducted eventually, and it was found that the patient lived in a farmhouse 20 days ago, and had eaten mutton soup in a local restaurant half a month before the onset of symptoms. Considering all the symptoms, such as fever, splenomegaly, and lymph nodes and the results of the blood culture positive for *Brucella*, the case was diagnosed as brucellosis. The biochemical identification confirmed *B. melitensis* biovar 3 (Table 2), which belongs to the dominant strain responsible for brucellosis in China. Then, the patient was given rifampin and doxycycline, and his fever gradually disappeared and his condition improved. He was discharged on May 21, 2019.

**Table 2:** Laboratory species-biovar identification results

Strain	Growth characteristics				Mono specific phage lysis					Species- Biovar
	CO_2_ requirement	H_2_S production	Thionin	Fuschin	Sera		RTD			
					A	M	Tb	Wb	BK_2_	
2019SC-YT	–	–	+	+	+	+	–	–	+	*Brucella melitensis* bv 3
544A	–	+	–	+	+	–	+	+	+	*B. abortus* bv 1
16 M	–	–	+	+	–	+	–	–	+	*B. melitensis* bv 1
Ether	–	–	+	+	+	+	–	–	+	*B. melitensis* bv 3
1330S	–	++	+	–	+	–	–	+	+	*B. suis* bv 3

*A* Anti-A serum, *M* Anti-M serum, *RTD* Routine Test Dilution, *Tb* Tb phage, *Wb* Wb phage, *BK*_*2*_ BK_2_ phage

## Follow-up

No clinical symptoms were observed after the patient was discharged, and rifampin and doxycycline were regularly taken. The standardized 6-week medication was prescribed. The patient continued to take medication for 3 months to avoid chronic infection. On September 15, 2019, the patient visited the local hospital in Yunnan for blood culture, and *Brucella* was not found. No abnormal symptoms were observed during the whole period of medication, proving that he was completely cured, and the medication was terminated.

## Discussion and conclusions

Brucellosis is an important zoonotic infection that remains to be a global public health issue. Currently, more than 170 countries have reported cases of brucellosis, with approximately 500 000 new cases reported each year [2]. Although it is well controlled in developed countries, it is a resurgent disease in developing countries. It is still an epidemic in the Mediterranean basin, the Middle East, Asia, Africa, Central America, and the Caribbean [9–11]. Brucellosis has been epidemic for many years in China. The epidemic situation showed an increasing trend in the mid-to-late 1990s, and this trend became more serious in the twenty-first century [12]. Currently, brucellosis in China is still mainly epidemic in the northern areas, but the epidemic intensity of brucellosis has increased in the southern areas, posing an important public health challenge in China [13].

At present, many methods have been adopted to detect brucellosis. Bacterial isolation remains the gold standard for testing [14]; however, currently used methods have low positive separation rate (less than 30%) [15], and the 7 days of culture is needed, and identification needs to be conducted in biosafety level 3 laboratories. Clinical laboratory personnel often suffer from laboratory-acquired infections due to the lack of effective personal protection. Compared with bacterial isolation, the serological diagnosis of brucellosis is relatively easy and rapid. For example, the screening test of rose bengal plate agglutination test (RBT) can be completed within four minutes, and the laboratory confirmed test of SAT can produce results about 20–22 h. The diagnostic accuracy is higher if the two approaches are used [16]. However, in recent years, the serological diagnostic technique has only been used in hospitals in the northern areas of China, such as Xinjiang and Inner Mongolia, which are high-epidemic areas, and serological diagnosis has not been promoted or widely applied nationwide. For example, in the case of the patient who was hospitalized in Chengdu Center for Disease Control and Prevention, blood culture was found only on day 7 after hospitalization. In view of the fact that there are reports of brucellosis in 31 provinces in China, and the epidemic intensity has increased in the southern areas [17], it is recommended that infectious disease and category A level III hospitals in the southern provinces of China be equipped with RBT and SAT reagents. RBT screening is recommended among patients with persistent fever and those who have been in contact with infected pig, cattle, and sheep and their products or pollutants, as this will greatly improve the sensitivity and timeliness of patient diagnosis and eventually facilitate a timely and effective clinical treatment, which will relieve the pain and disease burden of patients.

The treatment of brucellosis may be of great clinical importance in the management of HIV infection in a brucellosis-endemic country [6]. In this report, the patient was on ART to manage HIV, and doxycycline and rifampicin were regularly prescribed at the same time to treat brucellosis, and eventually treatment standards were achieved. This report is consistent with many other reports of AIDS complicated with brucellosis, demonstrating that brucellosis is completely curable as long as the medication is properly regulated [8, 18]. Bacterial infections are fairly common among HIV-infected patients due to the damage of the immune system [19]. This is because HIV infection is capable of inducing a state of profound immune dysregulation, which results in B cell hyperactivation and hypergammaglobulinemia [20, 21]. The other explanation for the observed higher prevalence of brucellosis infection in HIV patients is the defective cell-mediated and humoral immunities in HIV patients. HIV infection is associated with impaired production of antibodies against specific antigens and a loss of B cell memory [21]. Reduced levels of memory B lymphocytes in HIV-infected patients are correlated with defective humoral immunity and a source of abnormal IgG production in these patients [20]. This immune failure results in a higher rate of bacterial infections such as brucellosis. Consistent with this description, studies have reported more aggressive forms of brucellosis in immune-compromised and HIV-infected patients [22, 23]. Therefore, in provinces with high incidence of AIDS and brucellosis, such as Xinjiang and Henan, it is recommended to apply a joint-exam strategy to ensure the early detection, diagnosis, and treatment of patients to avoid progressing into chronic infection.

In summary, this is the first reported case of brucellosis concomitant with HIV infection in China. Brucellosis can be easily overlooked in infectious disease hospitals and in category A level III hospitals in the southern provinces of China; laboratories in these hospitals should be equipped with the reagents to diagnose brucellosis. Awareness of the clinical diagnosis of brucellosis should be improved among doctors in China. Meanwhile, it is suggested that provinces with a high incidence of AIDS and brucellosis implement a joint examination strategy model.

## Data Availability

All original (de-identified) data and materials are available upon request from the corresponding author.
